# Fibronectin Molecular Status in Plasma of Women with Endometriosis and Fertility Disorders

**DOI:** 10.3390/ijms222111410

**Published:** 2021-10-22

**Authors:** Jolanta Lis-Kuberka, Paulina Kubik, Agnieszka Chrobak, Jarosław Pająk, Anna Chełmońska-Soyta, Magdalena Orczyk-Pawiłowicz

**Affiliations:** 1 Department of Chemistry and Immunochemistry, Wroclaw Medical University, 50-369 Wrocław, Poland; 2 Hirszfeld Institute of Immunology and Experimental Therapy, Polish Academy of Sciences, 53-114 Wrocław, Poland; paulina.kubik@hirszfeld.pl (P.K.); chrobak@iitd.pan.wroc.pl (A.C.); soyta@hirszfeld.pl (A.C.-S.); 3 Clinical Department of Oncological and Procreative Gynecology of the 4th Military Clinical Hospital with the Polyclinic, 50-981 Wrocław, Poland; jpajak@4wsk.pl

**Keywords:** endometriosis, fertility disorders, molecular status of fibronectin, fibrin

## Abstract

The diagnosis of endometriosis and fertility disorders is difficult; therefore, it is necessary to look for reliable biomarkers. Analysis of the molecular status of fibronectin as a key player in repair and wound healing processes, as well as in coagulation and fibrinolysis pathways, is justified. ELISA and SDS-agarose immunoblotting were applied to determine the fibronectin concentration and presence and occurrence of soluble FN-fibrin complexes in the blood plasma of women with endometriosis (n = 38), fertility disorders (n = 28) and the healthy group (n = 25). The concentration of fibronectin in the blood plasma of women with endometriosis (292.61 ± 96.17 mg/L) and fertility disorders (287.53 ± 122.68 mg/L) was significantly higher than in the normal group (226.55 ± 91.98 mg/L). The presence of FN-fibrin complexes of 750, 1000, 1300, 1600 and 1900 kDa in the plasma of women with endometriosis and fertility disorders was shown. The presence of FN-fibrin complexes with a molecular mass of more than 1300 kDa in women with endometriosis and infertility and the complete absence of these complexes in healthy women may indicate an increased and chronic activation of coagulation mechanisms in these patients. The presence of complexes of high molecular mass may be one of the biomarkers of fertility disorders in women.

## 1. Introduction

In recent years, the rapid development of civilization diseases has contributed to a significant increase in incidents of fertility problems in women [1,2]. Number of factors are involved in the development of infertility and the most common are hormonal imbalances/disorders [3,4], age [2,5], abnormal body weight [4,6], and certain diseases, including polycystic ovary syndrome [7] and endometriosis [6].

Endometriosis (endometrial fibrosis) is a gynecological disease defined as the presence and proliferation of endometrial-like tissue outside the uterine cavity, which induces chronic inflammation leading to the formation of scars and adhesions mainly in the reproductive system but also in the peritoneum and other organs of the body. It ultimately may become a direct cause of women′s infertility [8,9,10,11,12]. Endometriosis affects up to 10% of women in their reproductive age and due to its chronicity, substantially affects their health-related quality of life [13]. In addition to that, the occurrence of endometriosis is associated with dysmenorrhea, dyspareunia, gastrointestinal problems, fatigue, headaches, lower back, and abdominal pain [14]. In the reproductive tract, endometriotic lesions are the most commonly found on the ovaries and in fallopian tubes [15]. Cells in ectopic tissue are sensitive to ovarian hormones such as estrogen and progesterone, which regulate endometrial tissue growth by stimulating and inhibiting cell proliferation, respectively [16]. Due to the low specificity of endometriosis symptoms, its diagnosis is long-lasting and often causes a delay of up to seven to 10 years [17,18]. 

Despite many years of research, the pathogenesis of endometriosis was not fully examined and presented results are inconsistent. The fine-tuning of several matrix metalloproteinases (MMPs) and their inhibitors have been reported both in eutopic endometrium and endometriotic lesions, definitively demonstrating the extracellular matrix remodeling [19]. On the other hand, it was reported [20] that, during the menstrual cycle, the pattern of expression of some components of the extracellular matrix (ECM) (e.g., collagen IV, laminin, vitronectin and fibronectin) was similar in the analyzed cohort study groups, namely endometrial and endometriosis tissue samples. However, endometrial fibrosis, associated with excessive accumulation of ECM components [20] is observed around endometriotic outbreaks and possibly represents massive tissue inflammation and remodeling [21,22,23,24]. In response to damage, the chemokine signaling pathway crucial for repair and healing processes is activated [25,26,27].

Many factors influence the phenotypic presentation of endometriosis, namely the complex correlation between the genetic profile and hormones related to menstruation, as well as immune cells and inflammatory factors [28,29,30]. Formed outside the uterus, endometrial tissue is under immune surveillance, which translates into chronic inflammation [29]. Immune factors produced by macrophages and peritoneal neutrophils support endometrial cell growth and invasion of endometrial cells and angiogenesis [29,31]. Furthermore, simultaneously an imbalance of T cell subtypes results in an amended cytokine profile, the appearance of inflammation and finally affects the growth of endometriosis lesions [28].

One of the key players of the extracellular matrix, involved in biological processes, such as coagulation [32], fibrinolysis [33], tissue repair and remodeling [33,34,35,36,37], is fibronectin (FN), which has the ability to create natural connections with proteins, including fibrin (a common acute phase protein) [38,39]. FN is synthesized by hepatocytes and released in compact soluble globular dimer form into the blood as plasma FN (pFN), while insoluble cellular FN (cFN) is produced locally in tissues, predominantly by fibroblasts, endothelial cells and also by chondrocytes and myocytes [40,41]. Under physiological conditions, there are two major forms of fibronectin (pFN and cFN) in balanced amounts [41]. However, during tissue injury and/or pathological state, the levels of cFn and pFN are pruned to change and the equilibrium is disturbed. It was reported that the participation of particular forms can differ during wound healing, namely pFN [35] is important for the early stage, while cFN for the late stage of this process [36,37,42]. Furthermore, prolonged tissue damage is reflected in the accumulation of the cellular form of FN, which consequently may lead to the next damage [41]. It was showed that the level of fibronectin was significantly lower in the homogenates of endometrial tissue, and the mRNA expression was endometriosis stage-related and, as pointed out by Holzer et al., the reproductive performance of women suffering from endometriosis may be affected by the dysregulation of the FN1 gene [43]. On the other hand, aberrant activation of some genes (e.g., FN1) [43,44] and certain enzymes, such as NADPH oxidase [44], superoxide dismutase and glutathione peroxidase [45], are important players in inflammation-related diseases, including endometriosis. Increased expression of the fibronectin gene [44] and stimulation of the coagulation cascade by inflammatory agents (conversion of fibrinogen into fibrin) favor interaction of pFN and fibrin, which is reflected in the formation of the soluble supramolecular FN–fibrin complexes. Until now, such FN-fibrin complexes have been detected in plasma from patients with some pathological conditions [46,47,48,49], in human puerperal and cord plasma [50], and additionally in an animal model (i.e., in dams of stillborn dairy calves [51] and in plasma from the cynomolgus monkey after treatment with recombinant clotting factor XIII) [52].

As previously reported, plasma FN-fibrin complexes have shown prerequisite prothrombotic activities, support platelet aggregation, thrombus growth and stability [32,37,53,54], and are considered a self-limiting regulatory factor in protecting against hemorrhage and excessive thrombus formation, as well as vessel occlusion. Furthermore, soluble supramolecular FN-fibrin complexes are crucial for fibrin clot and participate in the creation of a provisional matrix that results in improved cell adhesion and migration during repair processes [34,42]. In light of the above, hemostasis disorders, activation of the coagulation system and repair processes involving FN molecular forms may accompany the formation of endometriotic lesions formation, their development, and healing. Our objective was to analyze molecular status of fibronectin, namely the concentration of plasma FN and the occurrence of soluble supramolecular complexes of FN-fibrin, in plasma of women with endometriosis and fertility disorders to evaluate fibronectin molecular forms as a potential clinical biomarker.

## 2. Results

### 2.1. Characteristics of the Study Population

**Figure 1:**
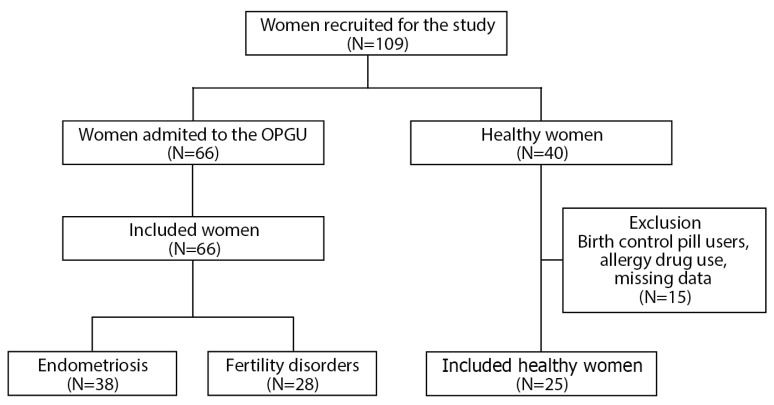
Flow chart of the study.

Ninety-one blood plasma samples were collected from women with: endometriosis (n = 38), fertility disorders of (n = 28), and healthy women (n = 25) (Figure 1). The detailed characteristics of the study population are shown in Table 1. The age of the women for the group of endometriosis, fertility disorders, and normal was 32.7 ± 5.3, 35.2 ± 3.7 and 27.0 ± 5.2 (*p* < 0.0008), respectively, and the BMI reached the value of 21.7 ± 2.7, 24.3 ± 4.1 and 22.4 ± 2.7 kg/m2 (*p* < 0.01), respectively.

Women with experience of miscarriage constitute 18.4%, 7.2% and 0%, respectively, for the group of endometriosis, fertility disorders, and healthy women. In analyzed groups, namely endometriosis, fertility disorders and normal group, 13.2%, 50.0% and none of the women suffered from hypothyroidism, while insulin resistance was observed for 7.9%, 32.1% and 0% of the women in the cohort group, respectively. Polycystic ovary syndrome was recorded for 2.6% and 10.7% of the cohort group of endometriosis and fertility disorders.

### 2.2. Concentration of Total Plasma Protein

Total protein concentration in blood plasma samples did not differ significantly between groups, and amounted to 62.16 ± 12.93, 59.72 ± 9.92, and 60.97 ± 7.21 g/L in patients with endometriosis, fertility disorders and in the normal group, respectively (Table 2).

### 2.3. Concentration of Fibronectin

**Figure 2:**
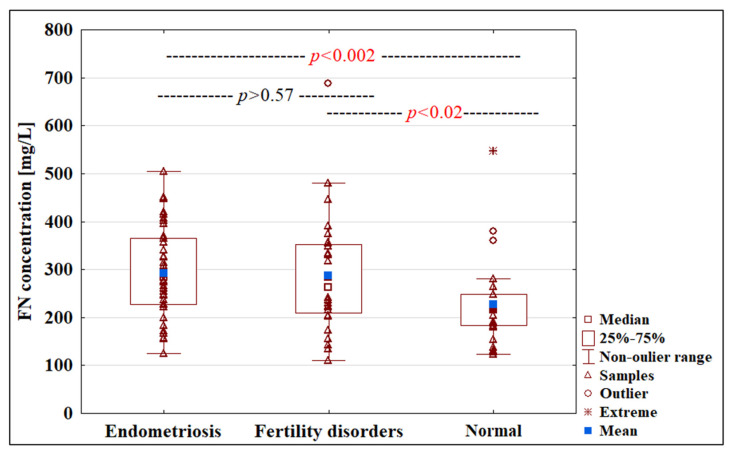
Box plot illustrating the FN concentrations in women’s plasma with endometriosis, fertility disorders and normal groups. The FN concentration were determined as described in Materials and Methods. Data are given as mean values, median and (25th and 75th) quartiles.

The concentration of FN in blood plasma samples from women with endometriosis (292.61 ± 96.17 mg/L) and fertility disorders (287.53 ± 122.68 mg/L) was significantly higher compared to the normal group 226.55 ± 91.98 mg/L (*p* < 0.002 and *p* < 0.02, respectively) (Table 2, Figure 2). 

Additionally, the concentration of FN in blood plasma samples for endometriosis, fertility disorders, and healthy women groups showed no correlation with age and BMI index (data not shown).

### 2.4. FN Molecular Forms Revealed by FN Immunoblotting 

**Figure 3:**
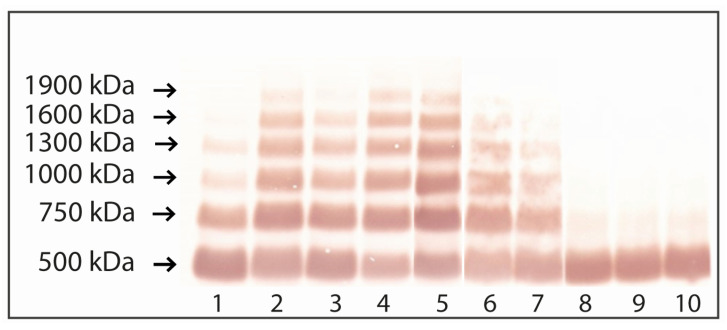
Representative immunopatterns of FN-fibrin complexes in blood plasma of women with endometriosis, fertility disorders and in the normal group.The 66 blood plasma samples of women with endometriosis and fertility disorders and 25 plasma samples from healthy individuals were subjected to SDS-agarose immunoblotting under non-reducing conditions [50,55]. Samples: Lanes 1–4 plasma of women with endometriosis; lanes 5–7 plasma of women with fertility disorders; lanes 8–10 plasma of women from control group. The molecular masses of plasma FN-fibrin complexes of 750 to 1900 kDa and 500 kDa FN dimer are shown by arrows on the left.

FN immunoblotting after SDS-agarose electrophoresis (Figure 3) showed, apart from the FN dimer (500 kDa), the presence of supramolecular FN-fibrin complexes with different frequencies. On the other hand, only in a healthy group of women a slight amount of FN monomer and the products of FN degradation (~2% of all FN forms) were observed. The relative amount of FN dimer was significantly lower in endometriosis (49.45 ± 22.19%, *p* < 0.000001) and in patients with fertility disorders patients (43.43 ± 22.32%, *p* < 0.000001) than in the normal group (84.80 ± 18.88%). Simultaneously, FN-fibrin complexes with molecular masses ranging from 750, 1000, 1300, 1600 to 1900 kDa, which were assigned as complexes I–V, respectively, and their total relative amount in groups of endometriosis and fertility disorders, constituted 50.55% and 56.57%, respectively, of all molecular forms, while in the normal group, 13.24% only (Figure 3, Table 2).

### 2.5. Frequency of Occurrence and Relative Amounts of Plasma FN-Fibrin Complexes

Frequency of occurrence/appearance and relative amounts of soluble plasma FN-fibrin complexes in endometriosis and fertility disorders groups were significantly higher than in normal group (Table 2).

For FN-fibrin complexes I–V (750–1900 kDa) (Table 2), the frequency of occurrence and relative amount decreased with increasing molecular mass of the complex in the endometriosis and fertility disorders groups.

The FN-fibrin complex I with molecular mass 750 kDa was revealed in samples of endometriosis, fertility disorders and normal groups (Table 2). The relative amount of FN-fibrin I complex was at a similar level in endometriosis (29.75 ± 6.88%) and fertility disorders (30.20 ± 5.58%), and was significantly higher (*p* < 0.00002 and *p* < 00006) in relation to the normal group (12.95 ± 15.75%). Significantly higher relative amounts of the complexes of FN-fibrin II (1000 kDa), III (1300 kDa) and IV (1600 kDa) were found in the endometriosis and fertility disorders group compared to the normal group (Table 2). The frequency of occurrence of FN-fibrin complexes was as follows: For 1000 kDa—0.61 and 0.75, for 1300 kDa—0.55 and 0.64 and for 1600 kDa—0.45 and 0.42, for the groups of endometriosis and fertility disorders, respectively.

The relative amount of the 1000 kDa complex was similar for plasma samples from women with endometriosis and fertility disorders (10.70 ± 9.60% and 14.69 ± 11.61%, respectively), and significantly higher (*p* < 0.00005 and *p* < 0.000001) in comparison with the normal group (0.29 ± 1.44%). The relative amounts of the FN-fibrin complex III and IV (1300 and 1600 kDa) were 6.91 ± 6.86% and 2.94 ± 4.09%, respectively, in the endometriosis group, and 7.66 ± 7.02% and 3.38 ± 4.94%, respectively, in the fertility disorders group (Table 2).

The presence of the 1900 kDa FN-fibrin complex was revealed only for two out of 38 plasma samples from endometriosis and for four out of 28 plasma samples from fertility disorders groups, but was undetected in the normal group.

### 2.6. Receiver Operating Characteristics (ROC) Curve Analysis 

Receiver operating characteristic (ROC) curve analysis of plasma FN concentration and the relative amount of the FN dimer (500 kDa) and FN-fibrin complex (750 kDa) band in the endometriosis vs. normal groups identified parameters, with a sensitivity and specificity of 0.722, 0.868 and 0.805, respectively (Table 3). 

A similar AUC value was obtained for fertility disorders versus normal group, namely 0.676 for FN concentration, 0.904 for FN dimer (500 kDa) and 0.809 for FN-fibrin complex (750 kDa), respectively (Table 3).

**Figure 4:**
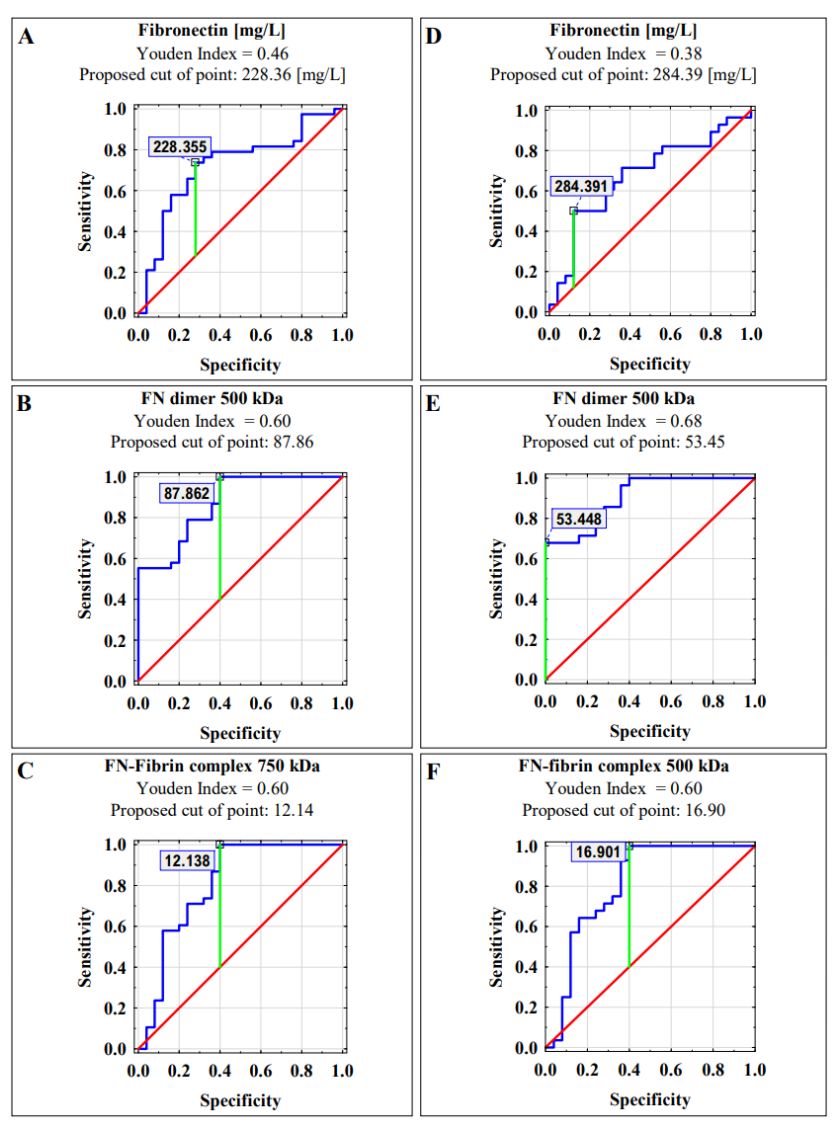
Receiver operating characteristic (ROC) curves for blood plasma FN concentration, FN dimer 500 kDa and FN-fibrin complex 750 kDa as markers of endometriosis (**A**–**C**) and fertility disorders (**D**–**F**). Data are given as area under the ROC curve (AUC) with a 95% confidence interval.

The Youden index method was performed to determine the determination of cut-off points (Figure 4). 

## 3. Discussion

The diagnosis of endometriosis, due to the low specificity of the symptoms, is long-term and remains challenging. Despite the wide range of research on nonsurgical detection of endometriosis, neither a single nor combined tool has been dedicated as a highly sensitive and specific diagnostic marker [1]. According to the above, new methods are constantly sought, and each new report regarding the pathogenesis of endometriosis [43,56,57,58] provides the opportunity for effective identification of women with endometriosis or other significant pelvic pathology and safe causal treatment.

To date, the number of studies that have examined fibronectin as a potential diagnostic tool in endometriosis has been limited [43,44,56]. According to scientific databases, such as the Web of Science and the National Center for Biotechnology Information, our findings are the first to characterize the molecular status of FN, namely, apart from FN concentration, the presence of supramolecular complexes from FN-fibrin in plasma of women with confirmed endometriosis and fertility disorders. Until now, the presence of FN-fibrin complexes has been reported for patients with other inflammatory diseases [46,47,48,49].

The interplay between the coagulation system and inflammation accompanies many diseases [59]. In the case of endometriosis, the chronic inflammatory process arises as a result of broken blood vessels. Cyclic angiogenesis associated with physiological menstrual cycles is especially important for growth as well as remodeling of the endometrium. Endometriotic lesions, to survive in their ectopic sites, require increased angiogenesis to provide an adequate blood supply [60,61]. However, as a result of vasculogenesis, endometriotic lesions-associated inflammatory reactions stimulate scarification processes [28,62]. Thus, the formation of FN-fibrin complexes seems to be essential in the creation of a fibrin clot and a provisional matrix, which promotes cell migration to the wound site and their adhesion [33,37,50,63,64]. The site of “endometriosis injury” becomes the local thrombin activation center and the state of hypercoagulability and hyperfibrinolysis accompanies women suffering from endometriosis [65]. Soluble FN-fibrin complexes support platelet aggregation and thrombus growth, and are presumed to act as a regulator in processes that ensure a balance between the occurrence of excessive hemorrhage and the formation of blood clots that cause obstruction of blood vessels [33].

It is believed that the presence of FN-fibrin complexes in plasma may be the result of the molecular relationship between inflammation and activation of the blood coagulation cascade. By activating the coagulation cascade, the inflammatory factor catalyzes the non-covalent interactions between the FN molecule and the αC domain of fibrin through the coagulation factor XIIIa. In subsequent stages, additional fibrin molecules with plasma FN and/or FN degradation products can attach to the FN-fibrin heterodimer, forming covalently cross-linked FN-fibrin complexes [50,55]. The quality and quantity of FN-fibrin complexes vary depending on the patient′s health status and can appear with the onset of some inflammation-related disease [46,47,48,49], as well as with physiological events such as delivery [50], and can disappear after patient’s recovery [55]. In the current study, the presence of FN-fibrin complexes was shown in the plasma of women with endometriosis and fertility disorders. In endometriosis, which accompanies prolonged menstruation and frequent breakthrough bleeding, the presence of FN-fibrin complexes in women’s plasma was expected, since they play an important role in restoring homeostasis. Parallelly, the presence of FN-fibrin complexes was also speculated for women who have problems with spontaneous conception, since low-grade chronic inflammation is associated with hypothyroidism [66], insulin resistance [67], obesity [68] and polycystic ovary syndrome [69], observed in 50.0%, 32.1%, 28.6% and 10.7%, respectively, of women with fertility disorders. In light of above, based on the detailed clinical examination of women included in our study, the influence of other disorders (including inflammatory diseases that are often associated with endometriosis and impaired fertility) on FN status and particular FN-fibrin complexes cannot be clearly excluded.

The frequency of complexes with molecular weights of ~750, ~1000 and ~1300 kDa for both analyzed groups (endometriosis and fertility disorders) was nearly 60% or greater. As reported previously, the FN-fibrin complexes with molecular masses 1000–1900 kDa were undetected in plasma of healthy women in reproductive age (i.e., 20–40 years old). However, in our cohort one sample was positive for the presence of 1000 kDa complex, probably due to the discreet imbalance of hemostasis that has not been reflected in the biochemical profile.

The obtained results may suggest that the endometriosis- and infertility-related inflammatory environment intensify the activation of the coagulation cascade which translates into the formation of soluble macromolecular FN-fibrin complexes. Such events, namely the occurrence of FN-fibrin complexes of increasing molecular weight, were previously confirmed [46,47,48,49,55] for different types and stages of inflammation, including the early stage of low-grade inflammation related to delivery [50]. Complex processes that occur during implantation of endometrial cells outside the uterus provoke an inflammatory response that leads first to low-grade inflammation [70,71,72]. However, further accumulation of cells with angiogenic potential has an impact on forming endometrial fibrosis [73,74,75,76] and finally leads to chronic inflammation. The important players in inflammation-related diseases are oxidative tissue factors that initiate molecular events of the coagulation system accompanied by FN. As a result of disturbance of the organism′s homeostasis caused by inflammation, the formation of FN-fibrin complexes may occur, with the aim of triggering molecular events leading to the restoration of balance in the functioning of the blood coagulation system. The FN-fibrin complexes formed in the subsequent steps of the coagulation process could be useful in shaping of the provisional matrix and remodeling during intensive repair processes [37,50,63,64].

The distribution of samples with successive stages of endometriosis in the study cohort is not equal; however, the statistical analysis did not show any correlation between the degree of advancement of endometriosis, namely stages I, II, III, and IV, and the quantity and quality of the present FN-fibrin complexes (data not shown). The low number of samples with I and II stages is related to the limited number of patients diagnosed with early endometriosis, where symptoms are often mild and patients are not included in laparoscopy assessment.

Women with initial stages of endometriosis, classified as stages I or II, do not have characteristic symptoms; menstruation pain is often mistakenly attributed to changes over the menstrual cycle. When the problem concerns women of reproductive age and spontaneous conception seems to be disturbed, patients come to an infertility clinic. Endometriosis is often diagnosed during routine procedures to determine the underlying cause of fertility problems. In light of this, the first group studied in this study was women with endometrial fibrosis and the second women without endometriosis but with fertility disorders. It should be noted that the appearance of soluble FN-fibrin complexes, in the early stage of gynecological disease, increases the diagnostic value of fibronectin molecular forms as a potential predictor of problems with fertility disorders (regardless of the presence of endometriosis) of women of reproductive age.

Despite statistically significant differences in the plasma′s FN concentration for the analyzed groups, the utility of this parameter as a predictor has limited clinical value. On the contrary, the use of immunochemical methods, such as sodium dodecyl sulfate (SDS) agarose immunoblotting, allowed the evaluation of molecular forms of FN and revealed their potential as diagnostic biomarkers. In the presented study, tested FN molecular forms, namely: The FN dimer (500 kDa) and the FN-fibrin complex 750 kDa reached moderate and high clinical values of AUC, 0.868 and 0.805, respectively, for women with endometriosis, and 0.904 and 0.0809, respectively, for women with fertility disorders (Table 3).

## 4. Materials and Methods

### 4.1. The Recruitment of Women

The study included women diagnosed with endometriosis and women with fertility disorders who were the patients of the Clinical Department of Oncological and Procreative Gynecology of the 4th Military Clinical Hospital with the Polyclinic (Wrocław, Poland). Women for the study were recruited for a protocol approved by the Ethics Committee of Wroclaw Medical University (No KB-407/2018) and informed written consent was obtained from all women. The age, BMI, number of births and miscarriages of the patients were recorded and a gynecological interview was performed.

Women with suspicion of endometriosis had undergone mainly laparoscopy procedures and, after histological verification, they were assigned to the endometriosis group. Additionally, women with endometriosis were classified according to the severity and extent of the disease according to the revised American Society for Reproductive Medicine (rASRM) classification [77].

The women in the fertility disorders group were unable to get pregnant for a period of 2 to 10 years. Among the recorded causes that might impact fertility were hormonal disorders (thyroid disease, hyperprolactinemia), uterine fibroids, polycystic ovary syndrome (POS), ovarian dysfunction, metabolic disorders (obesity, insulin resistance). The endometriosis was excluded in these women by medical interview, in a gynecological examination and in part of them in radical laparoscopy.

Exclusion criteria for women with endometriosis and fertility disorders were: cancer, HIV (human immunodeficiency virus) infection, jaundice and autoimmune diseases. Patients with thyroid disease, insulin resistance and polycystic ovary syndrome were not excluded. The normal group consisted of healthy women, the use of antiallergic drugs as well as birth control pill were exclusion criteria (Figure 1).

### 4.2. Blood Collection

Blood samples were collected from women with diagnosed endometriosis n = 38 and infertile disorders n = 28, and normal group n = 25, respectively. All blood samples were taken into tubes containing 3.2% sodium citrate as an anticoagulant.

### 4.3. Sample Pre-Treatment for Analysis 

Plasma samples were obtained by centrifugation of blood for 15 min at 2400× *g*, then the samples were aliquoted and frozen at −78 °C until analysis. Prior to assay, all samples were thawed for 1 h at 20–23 °C to allow solubilization of FN from possible aggregates.

### 4.4. Determination of FN Concentration

The concentration of fibronectin in plasma samples was quantified by enzyme-linked immunosorbent assay (ELISA) with a modified procedure published previously [50,78]. Briefly, anti-FN monoclonal antibodies (FN 30-8, Takara Shuzo Co., Ltd., Shiga, Japan) were used for coating of microtiter plate wells (Nunc International, Naperville, IL, USA). For determination of FN amount in samples, 100 μL of 4000-, and 8000-fold diluted in TRIS-buffered saline containing 0.05% Tween-20 (TBS-T, pH 7.5) blood plasma samples, and a standard preparation of FN from 1.56 to 50 ng/100 μL (Sigma, St. Louis, MO, USA) were transferred to the antibody-coated wells. Then, the HRP-conjugated detection antibody was added and the absorbency at 492 nm was measured.

### 4.5. Revealing of FN-Fibrin Complexes

Supramolecular bands of plasma FN-fibrin complexes were analyzed by SDS-agarose immunoblotting, as previously reported [50,55]. In brief: For horizontal slab SDS-agarose gel electrophoresis under non-reducing conditions, 300 ng of FN present in plasma samples were taken. The separated electrophoretically protein bands were capillary transferred onto a PVDF membrane (Bio-Rad Laboratories, Hercules, CA, USA) overnight. The anti-FN monoclonal antibody (FN 30-8, Takara Shuzo Co., Ltd., Shiga, Japan), as a detection antibody (Sigma, St. Louis, MO, USA), diluted 1:10 000 in 3% nonfat milk in TRIS-buffered saline (TBS), was taken and blots were incubated for 1 h at room temperature. As a secondary antibody, horseradish peroxidase-labeled rabbit anti-mouse immunoglobulins antibody (Sigma Chemical CO., St. Louis, MO, USA), diluted 1:5,000 in 3% nonfat milk in TBS, was taken and blots were incubated for 1 h at room temperature. The colored reaction was developed with a substrate solution containing 3′3-diaminobenzidine (Sigma, St. Louis, MO, USA) 0.1M citrate buffer, pH 6.0 with H_2_O_2_, DMSO, and blots were incubated for 3 min at room temperature. The reaction was stopped after 3 min, by removing the substrate solution and washing the blot using deionized water. For all washing steps, TRIS-buffered saline (TBS, pH 7.5) containing 0.05% Tween-20 was used.

To assign molecular masses of FN and FN-fibrin complex bands, plasma von Willebrand factor polymers [79] and standard plasma FN preparation (Sigma Chemical CO., St. Louis, MO, USA) were used. For confirmation of fibrin’s presence in the complex, rabbit antiserum to human fibrinogen (MP Biomedicals, Cappel, Santa Ana, CA, USA) was used as previously described [50].

After drying, immunoblots were scanned and FN bands were analyzed by densitometry [50] using myImage Analysis software version 2.0 (Thermo Fisher Scientific Inc., Waltham, MA, USA). Relative amounts of FN bands were expressed as the percentage of the total number of pixels in a lane.

### 4.6. Statistical Analysis

The statistical analysis was performed with TIBCO STATISTICA 13.3 (StatSoft, Inc., Tulsa, OK, USA). Data are presented as mean ± SD (standard deviation), median (25th-75th) and range. The normality of the distribution in relation to the variables was checked with the Shapiro–Wilk test. The chi-square test was used to compare the cohort/study population data. For analysis, nonparametric the Mann–Whitney U test was used for the calculation of statistical significance. A two-tailed *p*-value lower than 0.05 was considered significant. The diagnostic significance of the FN concentration and occurrence of FN-fibrin supramolecular complexes was assessed using receiver operating characteristic (ROC) curves.

## 5. Conclusions

Our study revealed that, in plasma of women suffering from fertility disorders (irrespective of the presence of endometriosis), soluble FN-fibrin complexes occur, aimed at triggering molecular events leading to the restoration of balance in the coagulation system and homeostasis. In light of the absence of FN-fibrin complexes in plasma from healthy women, the obtained results encourage further research on the use of the molecular status of fibronectin as a noninvasive diagnostic tool.

An unquestionable strength of the present study is the discovery of the presence of soluble FN-fibrin complexes of high molecular mass in the plasma of infertile women and patients with endometriosis. It may be a helpful tool in the non-surgical detection of fertility disorders, as well as endometriosis, and give promises to improve the early diagnosis of this heterogeneous clinical problem. On the other hand, some limitations should be considered when interpreting the findings of our study. Firstly, the influence of other disorders often associated with endometriosis and impaired fertility, such as hypothyroidism, insulin resistance, obesity and polycystic ovary syndrome, on FN status and formation of FN-fibrin complexes cannot be clearly excluded. Secondly, the age of participants of the control group was lower than for endometriosis and fertility disorder groups. However, due to the COVID-19 pandemic, it was difficult to collect samples from healthy women over 30 years old without any chronic diseases and hormonal contraception. Nevertheless, in our opinion, this has no significant impact on the obtained results, because generally the control group and the study groups included women in the same period (i.e., reproductive age).

Among several molecular forms of FN, which were evaluated, the highest clinical value was revealed for the relative amount of dimer of FN (500 kDa), indicating that this parameter could be considered as a potential predictor in the field of fertility disorders. Although further studies are needed, these data may shed light on the relevance of the biological role of FN-fibrin complexes in endometriosis and help in understanding the molecular mechanisms involved in the development of endometriosis and/or fertility disorders. However, at this stage of research, it is too early to attempt any changes in treatment strategies or additional options for the analyzed problems.

## Figures and Tables

**Table 1:** Characteristic of the study population.

	Endometriosis N = 38 (% (n/N))	Fertility Disorders N = 28 (% (n/N))	Normal Group N = 25 (% (n/N))	Chi-Square Test χ^2^	*p*-Value
Race/ethnicity white Europeans	100% (38/38)	100% (28/28)	100% (25/25)	NA	NA
Women′s age				22.8	0.0008
(mean ± SD)	32.7 ± 5.3	35.2 ± 3.7	27.0 ± 5.2		
20–29	26.3% (10/38)	3.6% (1/28)	80.0% (20/25)		
30–34	34.2% (13/38)	50.0% (14/28)	4.0% (1/25)		
35–40	31.6% (12/38)	32.1% (9/28)	16.0% (4/25)		
40+	7.9% (3/38)	14.3% (4/28)	0.0% (0/25)		
Women′s BMI, kg/m2				16.7	0.01
(mean ± SD)	21.7 ± 2.7	24.3 ± 4.1	22.4 ± 2.7		
underweight (<18.5)	13.5% (5/37)	0.0% (0/28)	8.0% (2/25)		
normal weight (18.5–24.9)	78.4% (29/37)	71.4% (20/28)	72.0% (18/25)		
overweight (25–29.9)	8.1% (3/37)	10.7% (3/28)	20.0% (5/25)		
class 1 obesity (30–34.9)	0.0% (0/37)	17.9% (5/28)	0.0% (0/25)		
Parity				5.7	0.2
0	89.5% (34/38)	92.8% (26/28)	100.0% (19/19)		
1	2.6% (1/38)	7.1% (2/28)	0.0% (0/19)		
2	7.9% (3/38)	0.0% (0/28)	0.0% (0/19)		
Miscarriages				5.7	0.2
0	81.6% (31/38)	92.8% (26/28)	100.0% (19/19)		
1	15.8% (6/38)	3.6% (1/28)	0.0% (0/19)		
2	2.6% (1/38)	3.6% (1/28)	0.0% (0/19)		
Stages of endometriosis according to rASRM classification				NA	NA
I/II	23.7% (9/38)	0.0	0.0		
III/IV	76.3% (29/38)	0.0	0.0		
Hypothyroidism	13.2% (5/38)	50.0% (14/28)	0.0	NA	NA
Insulin resistance	7.9% (3/38)	32.1% (9/28)	0.0	NA	NA
Polycystic ovary syndrome	2.6% (1/38)	10.7% (3/28)	0.0	NA	NA

The table shows values which are given as the percentage of women in the given subgroup (n) in relation to all women (N) for whom the specific information was available.

**Table 2:** Frequency of occurrence and relative amount of FN-fibrin complexes in blood plasma from women with endometriosis and fertility disorders.

		Frequency of Occurrence and Relative Amount of FN Forms Mean Value of Relative Amount ± SD Median (25th–75th) Range			*p*-Value E vs. N Group	*p*-Value FD vs. N Group
Plasma FN Forms	No Band MW (kDa)	Endometriosis (E) N = 38	Fertility Disorders (FD) N = 28	Normal (N) N = 25		
Protein concentration [g/L]		62.16 ± 12.93 61.58 (55.03–67.26) 45.38–115.39	59.72 ± 9.92 60.52 (53.52–64.01) 36.93–84.04	60.97 ± 7.21 59.89 (57.73–65.81) 45.96–73.84	*p >* 0.82	*p >* 0.47
FN concentration [mg/L]		292.61 ± 96.17 283.91 (227.11–364.76) 125.40–505.46	287.53 ± 122.68 262.85 (209.98–352.33) 110.95–687.68	226.55 ± 91.98 216.00 (183.79–248.00) 124.00–546.58	*p* < 0.002	*p* < 0.02
FN monomer ± degradations fragments	∼220–280	Not detected	Not detected	0.12 (3/25) 1.96 ± 5.45 0.00 (0.00–0.00) 0.00–18.03	NA	NA
FN dimer	∼500	1 (38/38) 49.45 ± 22.19 43.15 (30.97–66.67) 17.96–87.86	1 (28/28) 43.43 ± 22.32 40.27 (26.11–68.00) 9.68–83.10	1 (25/25) 84.80 ± 18.88 100.00 (69.30–100.00) 53.67–100.00	*p* < 0.000001	*p* < 0.000001
FN-fibrin complexes	I ∼750	1 (38/38) 29.75 ± 6.88 32.08 (26.20–33.74) 12.14–39.25	1 (28/28) 30.20 ± 5.58 30.92 (26.87–33.86) 16.90–41.94	0.48 (12/25) 12.95 ± 15.75 0.00 (0.00–28.48) 0.00–45.24	*p* < 0.00002	*p* < 0.00006
	II ∼1000	0.61 (23/38) 10.70 ± 9.60 13.87 (0.00–19.41) 0.00–25.89	0.75 (21/28) 14.69 ± 11.61 16.21 (2.38–22.25) 0.00–48.39	0.04 (1/25) 0.29 ± 1.44 0.00 (0.00–0.00) 0.00–7.20	*p* < 0.00005	*p* < 0.000001
	III ∼1300	0.55 (21/38) 6.91 ± 6.86 7.29 (0.00–12.96) 0.00–18.72	0.64 (18/28) 7.66 ± 7.02 7.47 (0.00–13.21) 0.00–21.74	Not detected	NA	NA
	IV ∼1600	0.45 (17/38) 2.94 ± 4.09 0.00 (0.00–5.12) 0.00–15.92	0.42(12/28) 3.38 ± 4.94 0.00 (0.00–6.00) 0.00–17.39	Not detected	NA	NA
	V ∼1900	0.05 (2/38) 0.24 ± 1.09 0.00 (0.00–0.00) 0.00–6.12	0.14 (4/28) 0.65 ± 1.83 0.00 (0.00–0.00) 0.00–7.45	Not detected	NA	NA

Plasma FN forms were revealed by SDS-agarose immunoblotting (see Figure 1). The values for FN concentration are given as mean ± SD, median (25th to 75th) and range. Frequency of occurrence is the ratio of the number of samples containing the FN form to the total number of samples. In parentheses are given the number of samples which revealed the respective FN band. The relative amount of the FN band is the percentage of the total number of pixels found in the electrophoresis path and is expressed as mean value ± SD. The Mann–Whitney U test was used for statistical calculations, and a *p*-value lower than 0.05 was regarded as significant.

**Table 3:** Receiver operating characteristic (ROC) curves for plasma FN concentration and relative amount of dimer (500 kDa) and FN-fibrin complex 750 kDa as potential predictors of endometriosis and fertility disorders.

Parameter	AUC	AUC with 95% Confidence Interval (Lower–Upper)	Cut-Off Point	Sensitivity	Specificity	*p*-Value
Endometriosis						
FN concentration [mg/L]	0.722	0.590–0.854	228.36	0.7368	0.28	<0.001
FN dimer 500 kDa	0.868	0.781–0.956	87.86	1	0.4	<0.0001
FN-fibrin complex 750 kDa	0.805	0.681–0.929	12.14	1	0.4	<0.0001
Fertility Disorders						
FN concentration [mg/L]	0.676	0.528–0.824	284.39	0.5	0.12	<0.02
FN dimer 500 kDa	0.904	0.828–0.981	53.45	0.6786	0	<0.0001
FN-fibrin complex 750 kDa	0.809	0.681–0.936	16.90	1	0.4	<0.0001

Data are given as the area under the ROC curve (AUC) with a 95% confidence interval.

## Data Availability

Not applicable.
